# Correlations between maternal and neonatal serum selenium levels in full term neonates with hypoxic ischemic encephalopathy

**DOI:** 10.1186/s13052-015-0185-8

**Published:** 2015-10-28

**Authors:** Abdel-Azeem M. El-Mazary, Reem A. Abdel- Aziz, Ramadan A. Mahmoud, Mostafa A. El-Said, Nashwa R. Mohammed

**Affiliations:** Department of Pediatrics, Faculty of Medicine, Minia University, Minia, Egypt; Department of Pediatrics, Faculty of Medicine, Sohag University, Sohag, Egypt; Department of Clinical Pathology, Faculty of Medicine, Minia University, Minia, Egypt

**Keywords:** Selenium, Neonates, Hypoxic, Ischemic, Encephalopathy

## Abstract

**Abstract:**

Perinatal hypoxic-ischemic encephalopathy (HIE) is an important cause of brain injury in the newborn and can result devastating consequences. The principle mechanisms underlying neurological damage in HIE resulting from hypoxemia and/or ischemia is deprivation of glucose and oxygen supply which energy failure. A consequent reperfusion injury often deteriorates the brain metabolism by increasing the oxidative stress damage. Selenium is a constituent of the antioxidant enzyme Glutathione peroxidase and is vital to antioxidant defense. This study aimed to measure the serum selenium levels in full term neonates with HIE and their mothers and to correlate between them and the severity of HIE.

**Methods:**

The study included 60 full term neonates with HIE admitted to NICU of Minia university hospital during the period from January 2014 to February 2015. Twenty apparently healthy full term neonates selected as a control group. After history taking and careful clinical examination; all neonates were subjected to: Complete blood count, renal and liver function tests and serum electrolytes. Serum selenium was measured for all neonates and their mothers within 48 h of life using atomic flame spectrophotometer method.

**Results:**

Neonates with HIE had significant lower serum selenium levels than normal healthy neonates (*p* = 0.001**) with the lowest levels in neonates with severe HIE but there were no significant differences between patients and controls as regards the maternal serum selenium levels. Significant negative correlations between serum selenium levels and the severity of HIE and base excess were present, while positive significant correlations were present with Apgar score and pH. There were no correlations between serum selenium levels and maternal serum selenium levels urea or creatinine levels.

**Conclusions:**

Neonates with HIE had lower serum selenium level than normal healthy neonates which is not dependent on the maternal serum selenium levels and was negatively correlated with the severity of HIE.

## Background

Perinatal hypoxic-ischemic encephalopathy (HIE) is an important cause of brain injury in the newborn and can result in long-term devastating consequences [1]. The development of brain injury after hypoxic insult is an evolving process during the acute insult and extending into a reperfusion phase. The principle mechanism underlying neurological damage in HIE resulting from hypoxemia and/or ischemia is deprivation of glucose and oxygen supply which causes a primary energy failure and initiates a cascade of biochemical events leading to cell dysfunction and ultimately to cell death [2]. A consequent reperfusion injury often deteriorates the brain metabolism by increasing the oxidative stress damage with particular increase in extracellular glutamate, excessive activation of glutamate receptors (excitotoxicity), increase in cytosolic calcium (Ca^2+^) and generation of oxygen free radicals. Hypothermia is the treatment of choice in neonatal HIE at present [3]. Combined therapy of hypothermia and other adjuvant neuroprotective strategies focusing on prevention of acute injuries and enhancement of neural repair is expected to improve the neurological outcomes of HIE [4].

Selenium is a trace element of tremendous importance in human health. It is a constituent of the antioxidant enzyme Glutathione peroxidase and therefore is vital to antioxidant defense. Several diseases of the neonates have been shown to be caused at least in part by oxygen free radicals like neuronal injury of HIE, bronchopulmonary dysplasia, retinopathy of prematurity, necrotizing enterocolitis and patent ductus arteriosus. Good selenium nutrition is important for antioxidant defense in neonates [5].

### Aim of the work

This study aimed to measure the serum selenium levels in full term neonates with hypoxic ischemic encephalopathy and their mothers and to correlate between these levels and the grades of HIE according to Sarnat and Sarnat staging system.

## Methods

This study included 60 full term (≥37 weeks) neonates with HIE (group I) admitted to neonatal intensive care unit of Minia university hospital for children, gynecology & obstetrics after approval of hospital ethics committee & informed parents’ consents, during the period from January 2014 to February 2015. According to Sarnat & Sarnat staging system; the neonates of this group classified into 3 subgroups as follow: neonates with mild HIE (20 neonates), with moderate HIE (20 neonates) and with severe HIE (20 neonates). Twenty apparently healthy full term neonates delivered in Minia university hospital selected as a control group (group II) and matched in terms of age, weight, sex and gestational age with the study group.

The criteria for diagnosis of HIE according to American academy of pediatrics (AAP) were: Apgar score ≤ 3 for ≥10 min, disturbed conscious level, abnormal neuromuscular control in the form of (abnormal muscle tone, abnormal posture, abnormal stretch reflexes and presence or absence of segmental myoclonus), abnormalities in complex reflexes in the form of (suckling, Moro, oculovestibular and tonic neck), abnormal autonomic function (abnormal pupil size, abnormal respiration, abnormal heart rate, abnormal GIT motility) presence or absence of seizures, PH ≤ 7.0 and base deficit ≥ 16 mmol /L [6, 7].

Preterm neonates less than 37 weeks, neonates with sepsis, respiratory distress, jaundice, CNS infection as well as neonates with renal, cardiovascular, hepatic diseases, associated congenital anomalies were excluded from the study. Maternal diseases as hypertension, diabetes mellitus, cardiac, neurological diseases and anemic mothers were excluded from the study. After history taking and careful clinical examination; all neonates were subjected to the following laboratory investigations: Complete blood count (by Sysmex, Japan), urea and creatinine as well as serum electrolytes (sodium, potassium and ionized calcium) were measured (by fully automated clinical chemistry auto-analyzer system Konelab 20i), liver function tests (using Integra 400 auto analyzer). Another sample of venous blood was aspirated within the first 48 h of life from all neonates as well as their mothers to measure the serum selenium levels using atomic flame spectrophotometer method, hydride generation atomic absorption.

### Statistical analysis

Finally, data entry and analysis were all done with IBM compatible computer using Statistical Package for Social Science (SPSS) for windows version 19. Graphics were done using Excel. Quantitative data were presented by mean and standard deviation, while qualitative data were presented by frequency distribution. Correlation, Chi Square, Spearman’s test and *T* test were done. The probability of less than 0.05 was used as a cut off point for all significant tests.

## Results

**Fig. 1:**
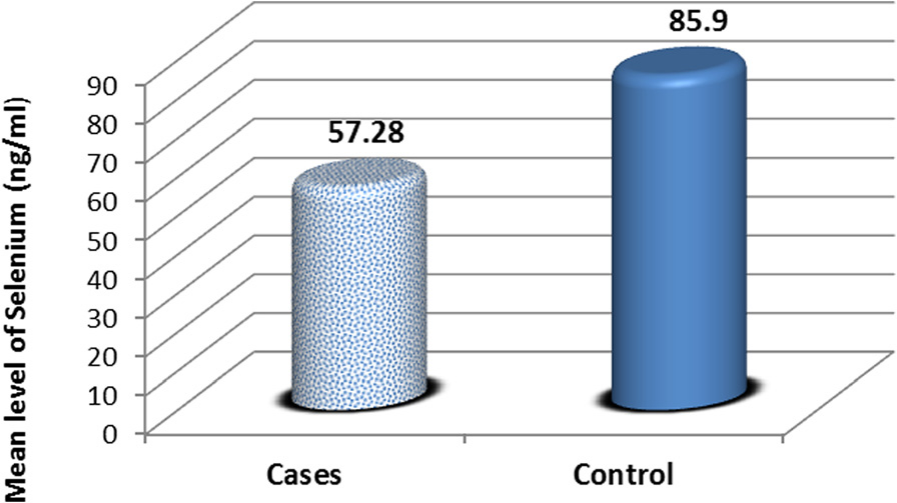
Serum selenium levels in cases and controls

Neonates with HIE had significant lower serum selenium levels (57.28 ± 37.20 ng/ml) than normal healthy neonates (85.9 ± 26.80 ng/ml) (*p* = 0.001**) with the lowest levels in neonates with severe HIE but there were no significant differences between patients and controls as regards the maternal serum selenium levels (110.4 ± 17.16 v 118.2 ± 20.11 ng/ml respectively) Table 1 & Fig. 1.

**Table 1:** Serum selenium levels in studied groups

Parameters		Patients	Controls	*P*- value
Maternal serum selenium (ng/ml)	Range	89.5–128	92–131	0.08
	Mean ± SD	110.4 ± 17.16	118.2 ± 20.11	
Neonatal serum selenium (ng/ml)	Range	10–90	60.8–105.6	0.001^b^
	Mean ± SD	57.28 ± 37.20	85.9 ± 26.80	
S. selenium in mild HIE (ng/ml)	Range	61–90	60.8–90.2	0.05
	Mean ± SD	82.25 ± 25.10	85.9 ± 26.80	
S. selenium in moderate HIE (ng/ml)	Range	45–70	60.8–105.6	0.001^b^
	Mean ± SD	60.50 ± 7.200	85.9 ± 26.80	
S. selenium in severe HIE (ng/ml)	Range	10–49	60.8–105.6	0.001^b^
	Mean ± SD	29.10 ± 18.815	85.9 ± 26.80	

^a^significant, ^b^highly significant

*S.* serum, *HIE* hypoxic ischemic encephalopathy

Neonates with HIE had lower levels of hemoglobin, platelets count, pH, sodium and ionized calcium levels than controls, but had higher levels of base excess, urea, creatinine, ALT, AST and potassium than controls Tables 2 and 1.

**Table 2:** Laboratory data of studied groups

Lab. parameters		Patients	Controls	*P* - value
		*N* = 60	*N* = 20	
Hb(g/dl)	Range	10–17	13.5–18	0.001^b^
	Mean ± SD	12.18 ± 1.62	15.67 ± 1.36	
Platelets/mm^3^	Range	100–430	200–450	0.01^a^
	Mean ± SD	259 ± 90.33	332.20 ± 64.29	
WBCs/mm^3^	Range	4.3–13	4–11.6	0.1
	Mean ± SD	6.71 ± 2.16	7.56 ± 2.02	
pH	Range	6.87–7.11	7.35–7.45	0.001^b^
	Mean ± SD	7.06 ± 0.18	7.38 ± 0.071	
Base deficit (mmol/L)	Range	11–20	11–15	0.001^b^
	Mean ± SD	16.93 ± 1.97	13.10 ± 1.252	
Urea(mg/dl)	Range	40–77	20–44	0.001^b^
	Mean ± SD	54.82 ± 7.92	32.95 ± 7.33	
Creatinine (mg/dl)	Range	0.8–2.5	0.5–1.5	0.001^b^
	Mean ± SD	1.863 ± 0.44	1.00 ± 0.35	
ALT(Unit/L)	Range	25–89	10–38	0.001^b^
	Mean ± SD	56.25 ± 17.05	27.45 ± 5.38	
AST(Unit/L)	Range	10–80	8–43	0.001^b^
	Mean ± SD	61.58 ± 17.769	26.0 ± 4.96	
Na (mmol/L)	Range	123–150	135–148	0.001^b^
	Mean ± SD	129.82 ± 7.40	139.09 ± 2.92	
K (mmol/L)	Range	3.8–7.9	3.5–5.3	0.001^b^
	Mean ± SD	6.82 ± 0.63	4.16 ± 0.53	
Ca (mmol/L)	Range	0.6–1.3	1–1.3	0.001^b^
	Mean ± SD	0.98 ± 0.18	1.17 ± 0.10	

^a^significan, ^b^highly significant

*Hb* hemoglobin, *WBCs* white blood cells, *ALT* alanine transferase enzyme, *AST* aspartate transferase enzyme, *Na* sodium, *K* potassium, *Ca* calcium

As regards the demographic data, there were no significant differences between neonates with HIE and controls as regards the neonatal age, gestational age, birth weight, mode of delivery and sex but they had significant lower Apgar scores at 1 and 5 min than normal healthy neonates Table 3.

**Table 3:** Demographic data and Apgar score of studied groups

Characters		Patients	Controls	*P* – value
		*N* = 60	*N* = 20	
Neonatal age (days)	Range	1–7	1–7	0.4
	Mean ± SD	3.57 ± 1.960	3.15 ± 2.033	
Gestational age (weeks)	Range	37–42	37–42	0.8
	Mean ± SD	39.08 ± 1.418	39.15 ± 1.387	
Birth weight (kg)	Range	2.7–4.5	2.9–4.3	0.2
	Mean ± SD	3.245 ± 0.1780	3.195 ± 0.1957	
Sex	Male	28 (46.7 %)	8 (40 %)	0.7
	Female	32 (53.3 %)	12 (60 %)	
Maternal age (years)	Range	19–38	18–33	0.7
	Mean ± SD	23.5 ± 11.4	22.4 ± 10.8	
Mode of delivery	Vaginal	26 (43.3 %)	8 (40 %)	0.8
	CS	34 (56.7 %)	12 (60 %)	
Apgar score at 1 min	Range	2–5	8–10	0.001^b^
	Mean ± SD	3.06 ± 1.40	8 ± 0.78	
Apgar score at 5 min	Range	3–7	8–10	0.001^b^
	Mean ± SD	4.15 ± 1.51	9 ± 0.89	

^a^significant, ^b^highly significant

**Fig. 2:**
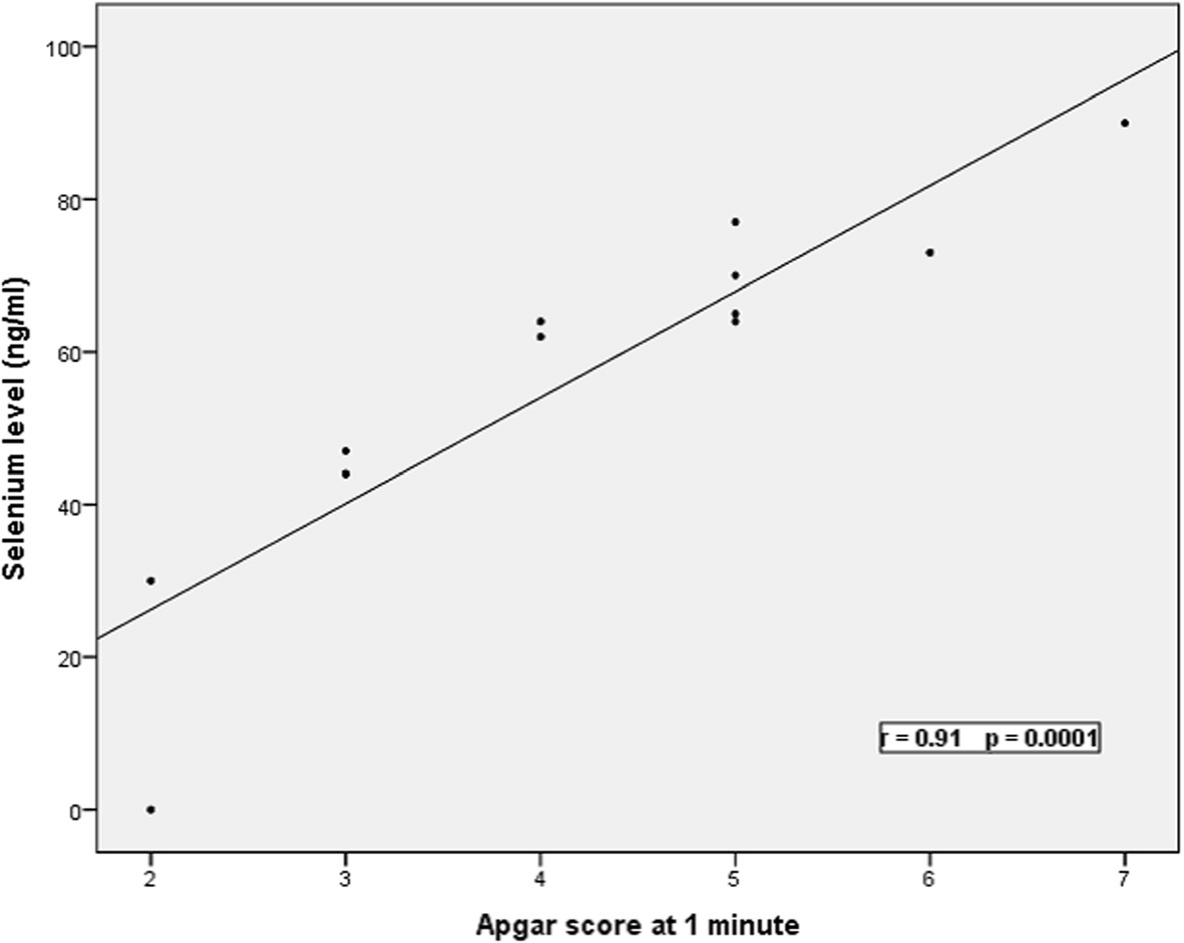
Correlations between serum selenium level and Apgar score at 1 min

**Fig. 3:**
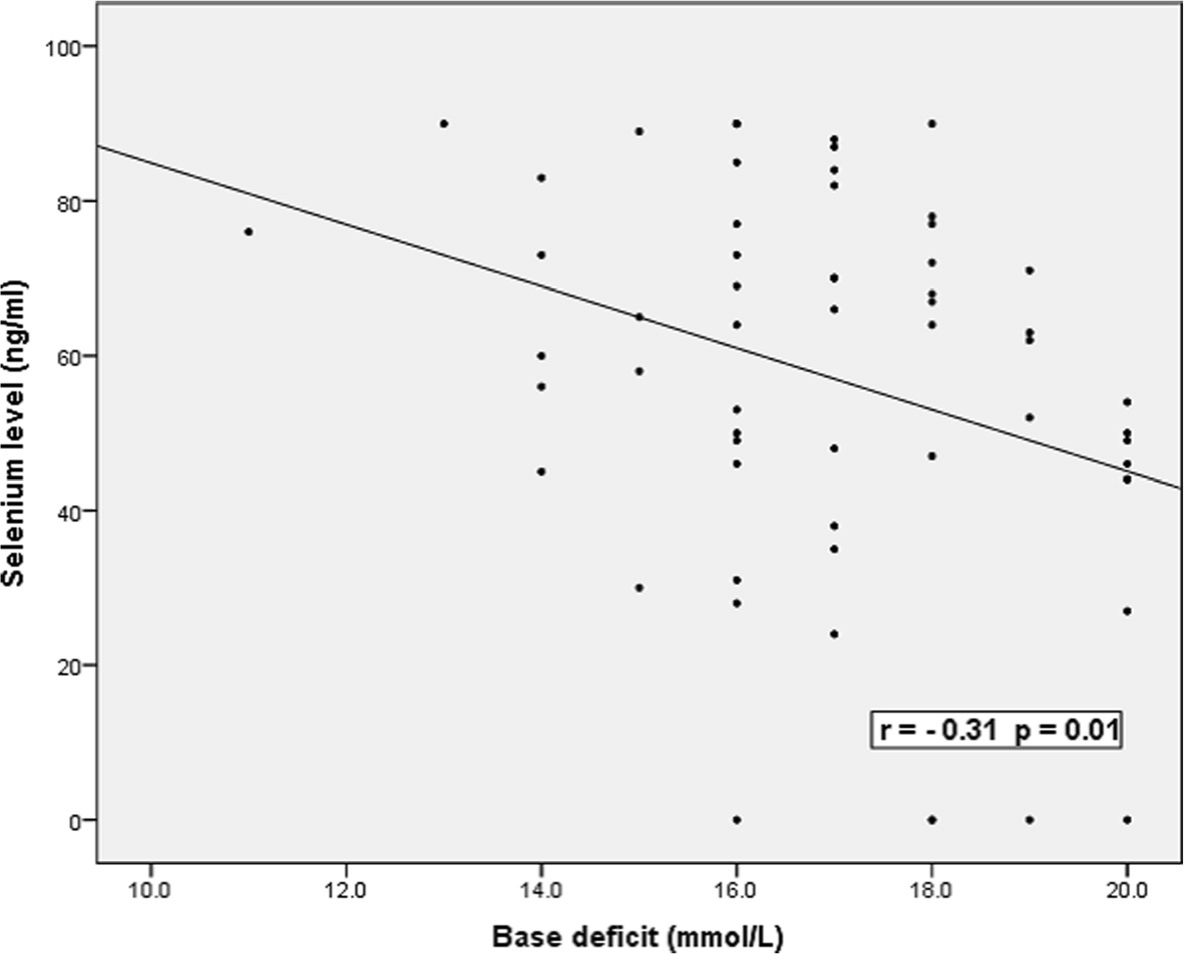
Correlations between serum selenium level and base deficit

Significant negative correlations between serum selenium levels and the severity of HIE and base excess were present, while positive significant correlations were present with Apgar score, pH, hemoglobin levels and platelets count. There were no correlations between serum selenium levels and maternal serum selenium levels, neonatal age, gestational age, weight, white cell count, urea or creatinine levels Table 4 & Figs. 2 and 3.

**Table 4:** Correlations between serum selenium levels and the studied parameters in patients group

Item	Serum selenium level	
	r	p
Neonatal age (days)	0.05	0.70
Gestational age (weeks)	0.18	0.1
Birth weight (kg)	0.01	0.90
Apgar score at 5 min	0.90	0.001^b^
Hb (g/dl)	0.43	0.001^a^
Platelets/mm3	0.76	0.01^a^
WBCs/mm3	−0.11	0.402
pH	0.29	0.04^a^
Base deficit (mmol/L)	−0.31	0.01^a^
Urea(mg/dl)	−0.08	0.49
Creatinine (mg/dl)	0.01	0.70
ALT(Unit/ L)	−0.30	0.01^a^
AST(Unit/L)	−0.49	0.001^a^
Na (mmol/L)	−0.01	0.92
K (mmol/L)	0.03	0.78
Ca (mmol/L)	0.38	0.002^a^

^a^significant, ^b^highly significant

*Hb* hemoglobin, *WBCs* white blood cells, *ALT* alanine transferase enzyme, *AST* aspartate transferase enzyme, *Na* sodium, *K* potassium, *Ca* calcium

## Discussion

Neonatal encephalopathy is a heterogeneous syndrome characterized by symptoms of central nervous system dysfunction in newborns. Neonates with encephalopathy may exhibit abnormal level of consciousness, seizures, tone and reflex abnormalities, apnea and feeding difficulties [8].

Selenium is a trace element of very importance in human health. It is a constituent of the antioxidant enzymes as Glutathione peroxidase and therefore is vital to antioxidant defense. Several diseases of the neonate have been shown to be caused by oxygen free radicals including retinopathy of prematurity, bronchopulmonary dysplasia, necrotizing enterocolitis and patent ductus arteriosus [9–11].

In this study neonates with HIE had significant lower serum selenium levels than normal healthy neonates (*p*-value = 0.001**) and those with severe HIE were the most affected group than neonates with moderate HIE and/or mild HIE.

Selenium which is nutritionally essential for humans is a constituent of more than two dozen sialoproteins that play a critical role in CNS protection from oxidative damage as well as during stressful conditions as infection, reproduction, thyroid hormone metabolism and DNA synthesis [12–14].

In our study there were no significant differences between patients and controls as regards the neonatal age, gestational age, birth weight, sex and mode of delivery except for Apgar scores at one and five minutes which were lower in patients than controls as expected and this is agreement with American academy of pediatrics (AAP), 1996 [15].

There were no significant differences in this study between neonates with HIE and controls as regards the maternal serum selenium levels as well as there were no significant correlations between neonatal serum selenium levels and maternal serum selenium levels and this means that the lower levels of selenium in neonates with HIE were not dependent on the maternal selenium levels.

These results are in agreement with Boskabadi et al., 2010 [16] who studied the relation between maternal and neonatal selenium levels and the effect of selenium supplementation during pregnancy on neonatal oxidative stress prevention and proved that maternal selenium supplementation during pregnancy was safe but was not associated with a significant change in the extent of oxidative stress in neonates which means that maternal serum selenium levels had no effect on oxidative stress which occurs during the pathophysiology of HIE.

Geeta et al., 2000 [17] reported that maternal age, parity and socio-economic status did not affect the plasma selenium levels; the results which support ours.

Moreover, neonates with HIE had lower levels of pH and higher levels of base excess secondary to anaerobic glycolysis and increased production of lactic acid (lactic acidosis) and these results were in agreement with other reports [1, 2, 15].

The suppressive effect of hypoxia and/or ischemia on the erythropoiesis as well bone marrow functions during the neonatal period was reported by many authors explaining the lower levels of hemoglobin and platelets count in neonates with HIE than healthy neonates [18].

Many endocrinal disturbances were reported secondary to hypoxia and ischemia during the neonatal period as pituitary, parathyroid and thyroid gland dysfunction and this could explain the lower levels of sodium and ionized calcium in neonates with HIE in this study [18–20].

The higher levels of urea, creatinine and potassium associated with lower sodium and ionized calcium levels in neonates with HIE than controls could be explained in the light of the injurious effect of hypoxia and ischemia on the renal functions secondary to hypo-perfusion and ischemia of renal tissues resulting in tubular and cortical necrosis as reported by others [19, 21].

As a part of multiple organ dysfunction, the elevated liver enzymes in neonates with HIE may be attributed to the effect of hypoxia and ischemia on the liver functions and these results were in agreement with other studies [19–22].

Selenium has an antioxidant properties and it prevents cell bodies from oxidation and keeps them biologically healthy as reported by Rayman, 2000 and 2012 [23, 24]. This may explain the negative significant correlations between serum selenium levels and the severity of HIE, base excess and elevated liver enzymes as well as the positive significant correlations between selenium and pH and Apgar score at five minutes.

Wirth et al., 2010 [25] reported that neuronal selenoprotein expression is required for interneuron development and prevents seizures and neurodegeneration, also he reported that depletion of selenium or mutation results in exacerbation of neuronal loss and dysfunction are two mechanisms responsible for decreased activities of selenoenzymes.

Likewise, genetic inactivation of all sialoproteins in neurons leads to progressive neurodegeneration and these results support our results regarding the role of selenium in neuronal cell protection.

In this study there were no significant correlations between serum selenium levels and the levels of urea and creatinine which are two important markers for renal damage and this means the lower levels of serum selenium levels are not dependent on the renal function as an excretory organ, i.e. the lower serum selenium levels are due to consumption of selenium and not due to increased secretion of it by kidneys.

As an integral part of the glutathione peroxidases and thioredoxin reductase selenium interacts with nutrients that affect cellular redox status. Selenium as glutathione peroxidase supports the activity of vitamin E (α- tocopherol) in limiting the oxidation of lipids. Further thioredoxin reductase maintains the antioxidant function of vitamin C by catalyzing its regeneration from its oxidized form dehydroascorbic acid [26].

Yousuf et al., 2007 [27] and Sarada et al., 2008 [28] demonstrated that selenium supplementation ameliorates hypoxia/ischemia-induced neuronal death *in vitro* and *in vivo* and reported that selenium could prevent glutamate and hypoxia-induced cell death and selenium pretreatment reduces glutamate-induced reactive oxygen species (ROS) production and preserves mitochondrial membrane potential, preserves mitochondrial respiration and complex activities.

Saad et al., 2014 [29] reported that the values of serum zinc, selenium, and erythrocyte glutathione peroxidase are significantly lower in children with refractory epilepsy than healthy controls and these results support our results regarding the role of selenium in neuronal cell functions.

Finally, we can attribute the lower serum selenium levels in neonates with HIE than controls in the light of that selenium was used as antioxidant to protect brain cells from injury by free radicals that were released due to brain hypoxia and its levels were inversely related to the degree of hypoxia and ischemia in them. The results which were in agreement with other studies reported that selenium has an important role in prognosis of hypoxic ischemic injury as it preserves mitochondrial function, stimulates mitochondrial biogenesis and reduces the infarct volume after focal cerebral ischemia [30].

Selenium significantly reduces reactive oxygen species (ROS) production in hypoxia through different complex mechanisms: the first mechanism by increasing the activity of antioxidant enzymes and protection of the mitochondrial functional performance by preserving the mitochondrial membrane potential [31, 32] and the second mechanism by regulating ATP production and activities of the mitochondrial respiratory chain complexes [33, 34]. Selenium-through the previous two mechanisms- inhibits the mitochondria-initiated cell death pathway and autophagy activation and thus improves the neuro-survival [34–36].

There were no correlations between serum selenium levels and maternal serum selenium levels, neonatal age, gestational age, weight or white cell count and this is in agreement with other studies mentioned before [34–36].

We excluded preterm neonates less than 37 weeks as well as neonates with sepsis from this study to exclude the effect of prematurity and infection on the serum selenium levels as reported by other studies [5, 14, 16, 37].

## Conclusion

Neonates with HIE had lower serum selenium levels than normal healthy neonates. This decrease in serum selenium level is not dependent on their maternal selenium levels. Selenium levels were negatively correlated with the severity of HIE according to Sarnat and Sarnat staging system. Selenium supplementation for those neonates may be a helpful adjuvant line of therapy with hypothermia in the future.
